# A Bacterial Parasite Effector Mediates Insect Vector Attraction in Host Plants Independently of Developmental Changes

**DOI:** 10.3389/fpls.2016.00885

**Published:** 2016-06-23

**Authors:** Zigmunds Orlovskis, Saskia A. Hogenhout

**Affiliations:** Department of Cell and Developmental Biology, John Innes Centre, Norwich Research ParkNorwich, UK

**Keywords:** plant–microbe–insect interactions, effector biology, floral development, parasite–host interactions, adaptation, host manipulation, insect behavior

## Abstract

Parasites can take over their hosts and trigger dramatic changes in host appearance and behavior that are typically interpreted as extended phenotypes that promote parasite survival and fitness. For example, *Toxoplasma gondii* is thought to manipulate the behaviors of infected rodents to aid transmission to cats and parasitic trematodes of the genus *Ribeiroia* alter limb development in their amphibian hosts to facilitate predation of the latter by birds. Plant parasites and pathogens also reprogram host development and morphology. However, whereas some parasite-induced morphological alterations may have a direct benefit to the fitness of the parasite and may therefore be adaptive, other host alterations may be side effects of parasite infections having no adaptive effects on parasite fitness. Phytoplasma parasites of plants often induce the development of leaf-like flowers (phyllody) in their host plants, and we previously found that the phytoplasma effector SAP54 generates these leaf-like flowers via the degradation of plant MADS-box transcription factors (MTFs), which regulate all major aspects of development in plants. Leafhoppers prefer to reproduce on phytoplasma-infected and SAP54-trangenic plants leading to the hypothesis that leafhopper vectors are attracted to plants with leaf-like flowers. Surprisingly, here we show that leafhopper attraction occurs independently of the presence of leaf-like flowers. First, the leafhoppers were also attracted to SAP54 transgenic plants without leaf-like flowers and to single leaves of these plants. Moreover, leafhoppers were not attracted to leaf-like flowers of MTF-mutant plants without the presence of SAP54. Thus, the primary role of SAP54 is to attract leafhopper vectors, which spread the phytoplasmas, and the generation of leaf-like flowers may be secondary or a side effect of the SAP54-mediated degradation of MTFs.

## Introduction

Parasite-induced changes of host bodies, including changes in host development and behavior, are viewed as extended phenotypes of parasite genes (Dawkins, 1982). The parasite genes modulate the host phenotype in a direction that will aid the propagation and spread of the parasite, but that can prove detrimental to the host (Dawkins, 1982, 1990). In some cases, parasite reprogramming of the host can lead to extreme changes in host development and behavior. For example, the severe limb malformations of frogs by trematodes of the genus *Ribeiroia* is thought to increase predation of the locomotion-impaired frogs by birds, which are essential for spread of the trematodes in the environment (Johnson et al., 2004). As well, rodents infected with *Toxoplasma gondii* change their behavior increasing the likelihood of predation by cats, which are the definitive hosts for *T. gondii* (Berdoy et al., 2000). However, mechanisms involved in parasite-mediated alterations of host phenotypes are mostly unknown limiting our ability to investigate if the modulations are adaptive (Poulin, 1995, 2013). Only a few parasite genes that orchestrate dramatic changes in host phenotype and behavior have been identified so far (Hoover et al., 2011; MacLean et al., 2011, 2014; Sugio et al., 2011a, 2014).

It is often unclear whether dramatic changes in host phenotype are adaptive or side effects (Dawkins, 1990, 2004; Poulin, 1995, 2013). Parasite genes cooperate with each other and interact with host genes generating variation in the extended phenotype depending on the combination of parasite/host genotypes (Dawkins, 1990). In addition, parasite genes may interfere with multiple host factors, some of which have multiple functions, therefore generating phenotypic side effects (Cezilly et al., 2013). For example, genes involved in plant or animal immune responses may also have roles in development and behavior; Toll-like receptors in Drosophila control development of body axis and defense responses to pathogenic fungi and bacteria (Lemaitre et al., 1996; Artero et al., 2003). Similarly, in plants, TEOSINTE BRANCHED1, CYCLOIDEA, PROLIFERATING CELL FACTORS 1 and 2 (TCP) and MADS-box transcription factors (MTFs) regulate major aspects of plant growth and organ development, but also regulate, for example, synthesis and downstream signaling of the plant hormones jasmonic acid (JA; Schommer et al., 2008; Immink et al., 2012) and salicylic acid (SA; Wang et al., 2015) that have roles in plant defense.

Phytoplasmas are phloem-limited parasitic bacteria that induce dramatic changes in the development of their plant hosts, including proliferation of stems (witch’s brooms), conversion of flowers into leaf-like structures (phyllody) and stunting and yellowing (Bertaccini, 2007). Phytoplasmas depend on sap-feeding hemipteran insect vectors for transmission (Weintraub and Beanland, 2006). For Aster Yellows strain Witches’ Broom (AY-WB) phytoplasma (‘*Candidatus*’ Phytoplasma asteris) it was found that infected plants are more attractive to the aster leafhopper *Macrosteles quadrilineatus*, which is the most important insect vector of AY-WB (Frost et al., 2011, 2013; Sugio et al., 2011a,b). The two AY-WB virulence proteins (effectors) SAP11 and SAP54 modulate *Arabidopsis thaliana* development and promote attraction and reproduction of insect vectors to phytoplasma-infected *A. thaliana* plants (MacLean et al., 2011, 2014; Sugio et al., 2011a,b). Effector SAP54 induces the production of green and indeterminate leaf-like flowers that resemble phyllody symptoms (MacLean et al., 2011). SAP54 acts by degrading specific MTFs via the 26S proteasome requiring SAP54 interaction with the 26S proteasome shuttle factor RAD23 (MacLean et al., 2014). Phyllody symptoms have been observed in a broad range of phytoplasma-infected crops and wild plant species and genes that have sequence similarities to AY-WB *SAP54* are found in diverse phyllody-inducing phytoplasmas worldwide, suggesting that SAP54 effectors may have important contributions to phytoplasma fitness (MacLean et al., 2011; Maejima et al., 2014).

Leafhoppers feed and lay eggs mostly on green vegetation, which is more abundant in leaf-like flowers than in normal flowers (MacLean et al., 2011). Moreover, *A. thaliana* RAD23 is required for the SAP54 mediated development of leaf-like flowers and also for leafhopper preference (MacLean et al., 2014). Therefore, we hypothesized that the formation of leaf-like flowers is a phytoplasma-induced extended phenotype to attract leafhopper vectors. Surprisingly, we found that the leaf-like flowers do not play a role in insect vector preference. We argue that parasite-induced phenotypes of hosts are not always adaptive, even if the developmental changes induced by parasite infections are obvious (leaf-like flowers) and there is evidence of a mechanistic link (dependence on RAD23) between the developmental change of the host (leaf-like flowers) and factors that directly impact parasite fitness (attraction of insect vectors that spread phytoplasmas).

## Materials and Methods

### Rearing of Insects

Phytoplasma-free colonies of *Macrosteles quadrilineatus* Forbes (Hemiptera: Cicadellidae) were maintained on pathogen-free oat plants (*Avena sativa*) in an aerated 50x50x50cm transparent plastic cage at 22°C, long day photoperiod (16/8-h light/dark), 48% humidity. Phytoplasma-infected colonies were reared on AY-WB-infected China aster (*Callistephus chinensis*) and lettuce (*Lactuca sativa*) under the same conditions as healthy insect colonies.

### Generation of Plants for Leafhopper Choice Assays

Generation of 35S:GFP-SAP54 and 35S:GFP transgenic *Arabidopsis* lines was done according to methods described in (MacLean et al., 2011). *A. thaliana ap1* and *lfy* mutant were obtained from NASC (ID: N6232, allele *ap1-12*; ID: N6228, allele *lfy-1*). The 35S:SVP lines were kindly provided by Martin Kater and described in Gregis et al. (2013). The *rad23* mutant lines were provided by Richard Vierstra and described in Vierstra (2009).

Non-flowering plants for insect choice experiments were sown on insecticide-free F2 compost soil (Levington, UK) and grown at 22°C, short day photoperiod (10/14-h light/dark) for 8 weeks. In contract, flowering plants were grown at 22°C, long day photoperiod (16/8-h light/dark) for 6 weeks. One week after germination transgenic lines 35S:GFP-SAP54 and 35S:GFP were sprayed twice (one-week interval) with herbicide Harvest^®^ (13.52% w/v glufosinate–amonium) following manufacturers recommendations (Bayer, Cambridge, UK). Four-weeks old plants were transplanted into 10 × 10 × 10 cm (*H* × *W* × *D*) plastic pots. Experiments involving pre-cut flowers, floral stems were removed by metal scissors 4 days prior to insect addition.

**FIGURE 1:**
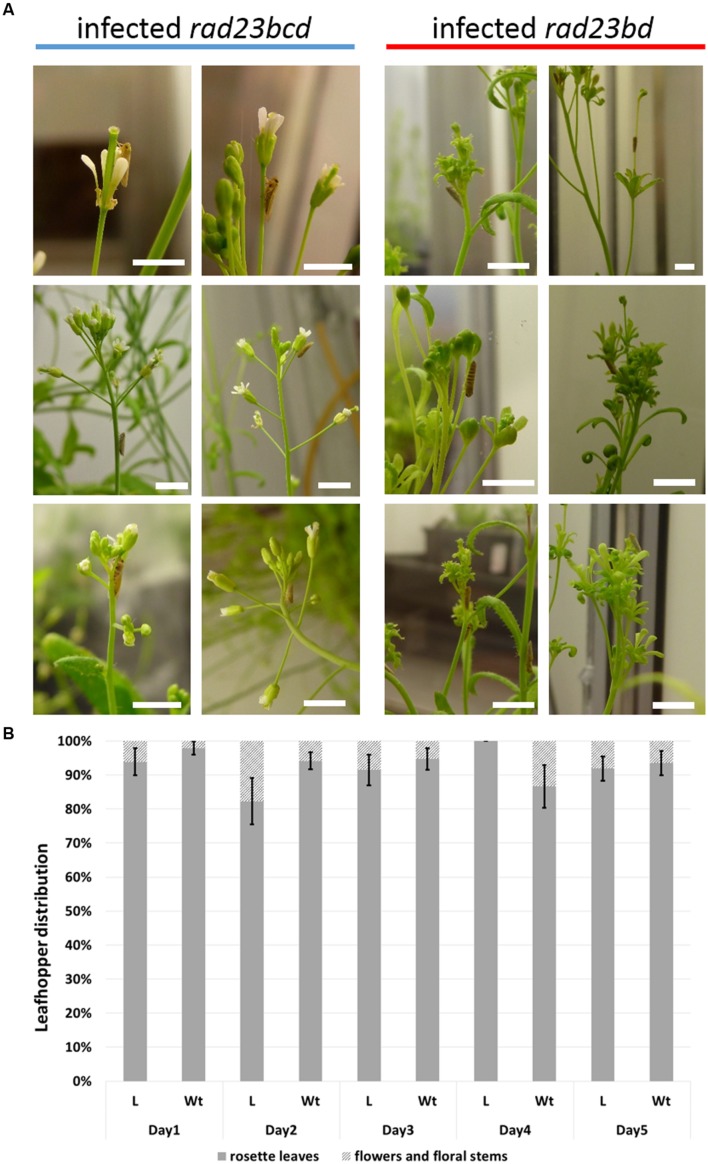
**Leafhoppers prefer vegetative plant tissues to flowers and have similar distribution on leaf-like and wild-type flowers.** **(A)** *Macrosteles quadrilineatus* leafhoppers were photographed whilst residing and feeding on all parts of infected *Arabidopsis thaliana rad23BD and rad23BCD* plants, including rosette leaves and petioles, stems, cauline leaves and flowers. Insects were found on carpels, sepals, petals and pedicels of wild-type-looking *A. thaliana* flowers as well as leaf-like flowers. White scale bars on each picture are 1 cm. **(B)** Number of insects found on rosettes or floral tissue is plotted as percentage of the total number of insects on wild-type plants and plants with leaf-like floral phenotype. Bars represent standard error of the mean of 8 independent replicate cages. *M. quadrilineatus* has significant residency preference for rosette leaves compared to other floral structures both on AY-WB infected *rad23BCD* mutant plants with leaf-like (L) flowers and AY-WB infected *rad23BD* mutant plants with wild-type flowers (GLM with time as covariate; *F*_1,137_ = 1797.78; *P* ≤ 0.001). There is no difference between insect residency on wild-type and leaf-like flowers during the five-day leafhopper choice experiment (GLM with time as covariate; *F*_1,67_ = 0.19; *P* = 0.666).

To generate infected plants (**Figure 1**), three-weeks old plants were infected with ‘*Ca*. Phytoplasma asteris’ strain AY-WB by adding five AY-WB-carrying adult *M. quadrilineatus* to each plant in a transparent perspex tube (10 cm high, diameter 4 cm) for 5 days. Two weeks after the removal of adult insects, three rosette leaves were collected for extraction of genomic DNA to confirm phytoplasma infection using AY-WB specific primers BF 5′AGGATGGAACCCTTCAATGTC 3′ and BR 5′ GGAAGTCGCCTACAAAAATCC 3′ (MacLean et al., 2014).

### Insect Choice Assays

**FIGURE 2:**
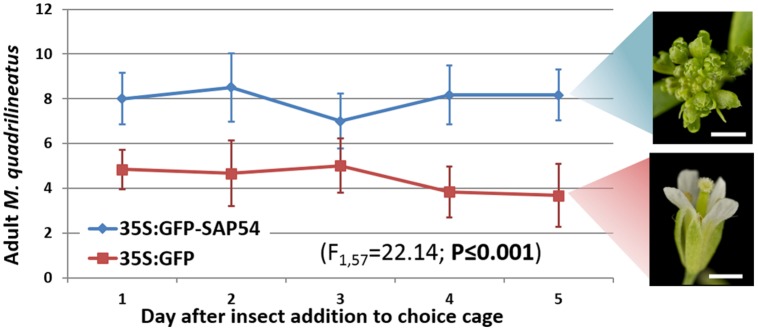
**Aster leafhopper *M. quadrilineatus* demonstrates greater residency preference for SAP54 expressing plants with leaf-like flowers.** More insects were found on SAP54 plants over the entire 5 day choice period (GLM with time as covariate; *F*_1,57_ = 22.14; *P* ≤ 0.001). Picture scale bar is 1 mm. Bars in the graph are one standard error of the mean.

**FIGURE 3:**
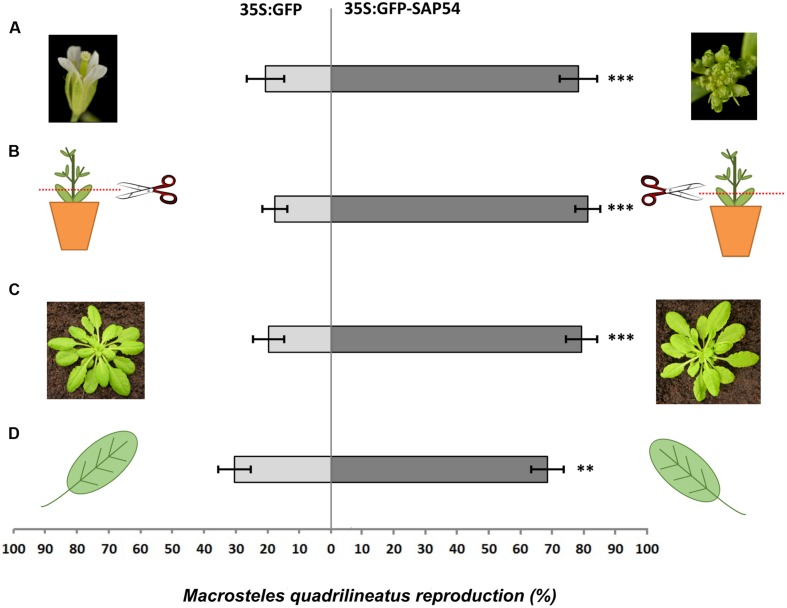
**Flowers and transition from floral to vegetative phase are not required for SAP54-mediated enhancement of insect colonization.** **(A)** *M. quadrilineatus* produces more nymphs on 35S:GFP-SAP54 transgenic *A. thaliana* (Col-0) plants with leaf-like flowers than on 35S:GFP (Col-0) control plants with wild type flowers. **(B)** Removal of flowers and floral stems does not affect *M. quadrilineatus* colonization preference of 35S:GFP-SAP54 transgenic *A. thaliana* (Col-0). **(C)** Leafhoppers also prefer GFP-SAP54 transgenic plants prior to transition from vegetative to floral growth. **(D)** *M. quadrilineatus* lays more eggs on single rosette leaves of GFP-SAP54 transgenic plants. Bars shown are one standard error of the mean. Each experiment was replicated in six independent choice cages. Significant difference in leafhopper reproductive preference are indicated as follows: ^∗∗∗^*p* ≤ 0.001; ^∗∗^*p* ≤ 0.025 (paired *t*-test). All experiments were repeated three times with similar results (**Supplementary Figure S1A**).

All insect choice experiments (**Figures 1**–**3** and **5**) were performed in transparent polycarbonate cages 62 cm × 30 cm × 41 cm (*H* × *W* × *D*). The opposite sides of the cage were fitted with white nylon mesh held by magnetic strips to the carcas of the cage for ventilation and access. GFP-SAP54 and GFP transgenic plants were placed randomly diagonally opposite each other in the corners of a cage. Ten male and ten female adult *M. quadrilineatus*, which did not carry AY-WB phytoplasma, were released from a transparent perspex tube (9 cm high, diameter 3 cm) in the center of the cage, at equal distance from each test plant. Adult insects were removed 5 days after addition to the cage. Plants were removed from the choice cage and contained individually in transparent perforated plastic bags at 22°C, long day photoperiod (16/8-h light/dark). Nymphs were counted on each test plant 14 days after removal of adult insects from the cages. Data were expressed as proportion of total number of nymphs found on the test plants within each choice cage. Similar experiments were done with phytoplasma-infected and healthy plants or wild type and MTF-mutants plants.

### Insect No-Choice Assay

**FIGURE 4:**
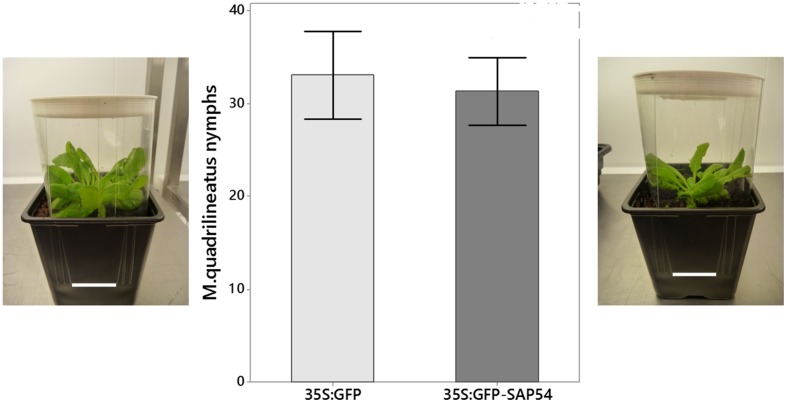
***Macrosteles quadrilineatus* leafhoppers produced similar nymph numbers when confined to 35S:GFP-SAP54 or 35S:GFP plants (no choice tests).** Leafhoppers were released on whole plants, which were caged inside a plastic tube as shown at left and right (picture scale bars are 5 cm). The middle graphs show mean numbers of leafhopper nymphs produced in these cages of three independent experiments (paired *t*-test; *n* = 6; *p* = 0.773). Bars shown are one standard error of the mean.

For the no-choice experiment (**Figure 4**) five female and five male non-infected adult *M. quadrilineatus* were added to individual plants surrounded by a transparent plastic cage. Plants were grown and insect progeny measured as in choice experiments.

### Single-Leaf Insect Choice Assays

For the experiment in **Figure 3D**, single rosette leaves that remained attached to GFP and GFP-SAP54 transgenic plants were fitted opposite each other in a 2 cm × 8 cm × 12 cm (*H* × *W* × *D*) transparent plastic cage fitted with nylon mesh-lined holes (4 cm diam.) to allow for air circulation. Five male and five female adult *M. quadrilineatus* leafhoppers (which did not carry AY-WB) were introduced into the cage and allowed free access to both leaves. Eggs were dissected and counted under stereomicroscipe (15×) 5 days after the first day of exposure to the insects.

### Statistical Analysis

Statistical analysis was performed in Minitab16. Insect oviposition data were analyzed using paired *t*-test, two-tailed *t*-test or GLM. Assumptions of the statistical tests – normal distribution and equal variance – were checked with the Anderson–Darling and the Levene’s tests, respectively.

## Results

The aster leafhopper *M. quadrilineatus* is the most important insect vector of the phyllody-inducing ‘*Ca*. Phytoplasma asteris’ strain AY-WB (Frost et al., 2011, 2013). This leafhopper favors the colonization of plants with leaf-like flowers (phytoplasma-infected *rad23bd* mutant plants and GFP-SAP54 transgenic plants) versus plants with wild-type flowers (phytoplasma-infected *rad23bcd* mutant plants and GFP transgenic plants; MacLean et al., 2014). These results prompted us to further examine if the insects are attracted by leaf-like flowers or repelled by wild-type flowers of phytoplasma-infected *rad23bd*/*rad23bcd* plants. Interestingly, leafhoppers resided and appeared to feed equally well on wild type and leaf-like floral structures (**Figure 1A**), suggesting that the insects do not prefer the leaf-like flowers to the wild-type ones. Moreover, we noticed, that most leafhoppers preferred to reside on the rosette leaves rather than floral stems or flowers (**Figure 1B**), suggesting that the flowers may not be required for leafhopper attraction. Nonetheless, we found that the insects spent more time on GFP-SAP54 transgenic plants with leaf-like flowers versus GFP transgenic plants with wild-type flowers (**Figure 2**), and these insects produced more progeny on GFP-SAP54 transgenic plants with leaf-like flowers (**Figure 3A**; **Supplementary Figure S1A**). However, when insects were not given a choice between host plants, by caging the leafhoppers on either GFP-SAP54 transgenic plants with leaf-like flowers or control GFP transgenic plants with wild type flowers, no increase in nymph production was observed (**Figure 4**). Thus, the observed leafhopper preference is the result of preferential orientation to plants with leaf-like flowers rather than an increase in reproductive efficiency of the leafhoppers on these plants.

To analyze the impact of leaf-like flowers on leafhopper preference further, we removed both the leaf-like and wild-type flowers from plants in the insect choice experiments and found that the leafhoppers then also preferred the GFP-SAP54 plants (**Figure 3B**; **Supplementary Figure S1A**), suggesting that the presence of leaf-like flowers are not required for leafhopper preference of GFP-SAP54 plants. *A. thaliana* plants used in insect choice tests so far were grown at long days to induce bolting and flowering. Next, we conducted choice tests on *A. thaliana* plants grown at short days that did not flower. Again, *M. quadrilineatus* produced more nymphs on GFP-SAP54 versus GFP (control) plants (**Figure 3C**; **Supplementary Figure S1A**), suggesting that insect preference for the GFP-SAP54 plants does not involve physiological and developmental transformations during floral transition. To confirm this finding, leafhoppers were also given a choice between single leaves of GFP-SAP54 and GFP plants. We found that the leafhoppers preferred to lay eggs onto single leaves of GFP-SAP54 plants (**Figure 3D**; **Supplementary Figure S1A**), indicating that leafhoppers are attracted solely to the leaves of GFP-SAP54 plants. Taken together, these data demonstrate that leaf-like flowers are not required for host plant selection by the leafhopper vector, and that SAP54 modulates processes in leaves to promote leafhopper attraction.

**FIGURE 5:**
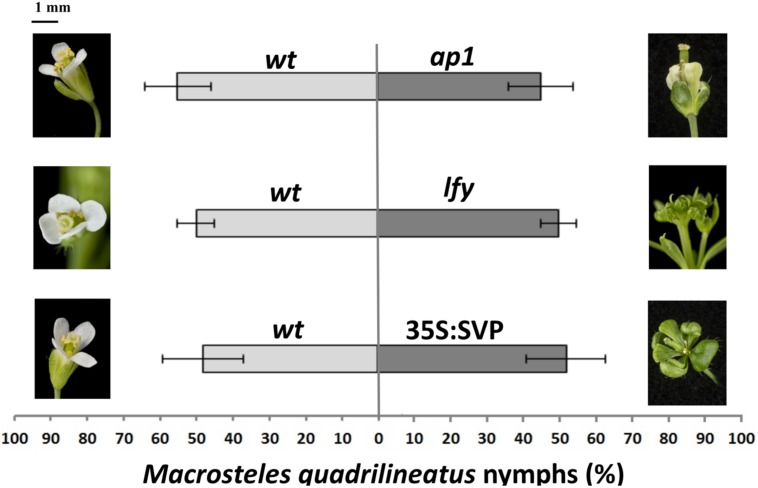
**Aster leafhopper *M. quadrilineatus* has similar oviposition preference for plants with normal and leaf-like flower phenotype that do not express *SAP54*.** *M. quadrilineatus* did not show a preference for colonization of Col-0 wild-type versus Col-0 apetala1 (*ap1*-12; *p* = 0.835), Col-0 versus Col-0 *leafy* (*lfy*-1; *p* = 0.985) and Col-0 versus 35S:SVP (Col-0; *p* = 0.960). Choice experiments were conducted with whole plants retaining both vegetative and floral organs. Data shown as percentage of *M. quadrilineatus* nymphs found on each test plant per total number of nymphs within a single choice cage (bars are standard error of the mean). Data were analyzed by paired *t*-tests. All experiments were repeated three times with similar results (Supplementary Figure S1B). Picture scale bar is approximately 1 mm.

The above experiments provide evidence that leaf-like flowers are not required for insect vector preference. Nonetheless, these flowers could contribute to the insect preference. To test this, we conducted choice experiments with *A. thaliana* lines displaying leaf-like flowers, including MTF mutant lines *ap1* (Mandel et al., 1992) and *lfy* (Weigel et al., 1992) and the 35S:SVP transgenic line (Gregis et al., 2013). All these lines produce flowers that share leaf-like structures reminiscent to those of phytoplasma-infected and GFP-SAP54 transgenic plants (MacLean et al., 2011). We found that leafhoppers produce similar numbers of progeny on both plants indicating no colonization preference for plants with leaf-like floral phenotypes (**Figure 5**; Supplementary Figure S1B) that is in agreement with our observation that leafhoppers make a choice based on the presence of rosette leaves only (**Figure 3D**). Thus, the leaf-like flowers are not required for leafhopper preference of GFF-SAP54 plants.

## Discussion

**FIGURE 6:**
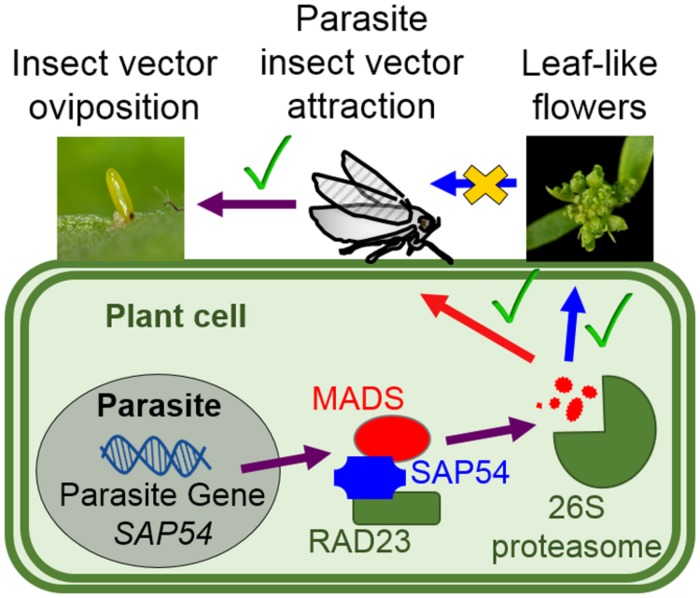
**Phytoplasma effector SAP54 mediates insect vector attraction to host plants independently of the presence of leaf-like flowers.** The phytoplasma virulence protein (effector) SAP54 interacts with specific MADS-box transcription factors (MTFs) and degrades these via the 26S proteasome leading to the development of leaf-like flowers that resemble those of phytoplasma-infected plants (MacLean et al., 2011, 2014). The SAP54-mediated degradation of MTFs is dependent on SAP54 interaction with the 26S proteasome shuttle factor RAD23 (MacLean et al., 2014). Leafhoppers prefer to lay eggs on SAP54 transgenic lines and phytoplasma-infected plants and this preference is also dependent on RAD23 (MacLean et al., 2014). Nonetheless, leaf-like flowers are not required for the leafhopper egg-laying preference. Whether MTFs that are degraded by SAP54 regulate other cellular processes, such as plant defense responses to insect pests, remains to be investigated.

Hitherto, direct analyses of the adaptive significance of parasite extended phenotypes have been limited because many parasites (such as phytoplasma) are not amenable to genetic manipulation and parasite genetic factors that induce the dramatic host alterations are often unknown. Given that leafhoppers feed and lay eggs mostly on vegetative tissues, including stems and leaves, and that the plant 26S proteasome cargo protein RAD23 is required for both the induction of leaf-like flowers and insect vector attraction (Weintraub and Beanland, 2006; MacLean et al., 2014), we hypothesized that leafhoppers may be attracted to leaf-like flowers of phytoplasma-infected and SAP54 transgenic plants. However, this study has shown that leaf-like flowers are not required nor are involved in attraction of the phytoplasma insect vectors. Moreover, leafhoppers preferred plant vegetative tissues above reproductive organs. Thus, leaf-like flowers do not promote leafhopper colonization, even though these two phenotypes are genetically connected via SAP54 interaction with the 26S proteasome cargo protein RAD23 (MacLean et al., 2014) (**Figure 6**).

Phyllody-inducing ‘*Ca*. Phytoplasma asteris’ phytoplasmas, such as AY-WB, often infect annual plants (Lee et al., 2004), which die upon flowering and seed production. Phytoplasmas are dependent on insect vectors for spread before plants die (Weintraub and Beanland, 2006). Hence, phytoplasmas that produce effectors, such as SAP54, which attract insect vectors are likely to spread faster than phytoplasmas that do not produce such effectors. Therefore it is likely that insect vector attraction is the extended phenotype of SAP54. Whether leaf-like flowers have a role in promoting phytoplasma fitness has become less clear. It is possible that the induction of phyllody is a side effect of SAP54-mediated modulation of processes involved in insect attraction. SAP54 induces leaf-like flowers by mediating degradation of MTFs via interaction with RAD23 (MacLean et al., 2014). MTFs are regulatory hubs for a plethora of physiological processes in plants, including plant immunity (comparable to animal HOX genes); several MTFs appear to (in)directly regulate cytokinin and JA synthesis and response genes (Gregis et al., 2013), which affect plant–insect interactions (Erb et al., 2012; Giron and Glevarec, 2014; Naessens et al., 2015; Schafer et al., 2015), such as that of the AY-WB leafhopper vector *M. quadrilineatus* (Sugio et al., 2011a). In addition, MTFs regulate age-related resistance responses to pests (Wilson et al., 2013). Therefore, SAP54-mediated degradation of MTFs may modulate plant immunity leading to attraction of the leafhoppers.

Another AY-WB phytoplasma effector, SAP11, binds and destabilizes specific members of the TCP family and promotes leafhopper oviposition activity in no-choice tests (Bai et al., 2012; Sugio et al., 2011a, 2014). TCPs are transcription factors that are conserved among plants and are regulatory hubs for plant growth and organ formation. In addition, TCPs regulate a variety of microRNAs and the plant defense hormones JA (Schommer et al., 2008; Immink et al., 2012) and SA (Wang et al., 2015). Another phytoplasma effector, Tengu, also induces witch’s broom-like symptoms in plants (Hoshi et al., 2009) and alters the plant JA and auxin hormone balance (Minato et al., 2014), and it was suggested that the witch’s broom-like symptoms attract the leafhopper vectors (Hoshi et al., 2009). However, given that SAP11 decreases JA production, which increases leafhopper colonization (Kallenbach et al., 2012; Lu et al., 2014; Sugio et al., 2011b), it remains to be investigated if witch’s broom symptoms are involved in the leafhopper colonization preference. Thus, the SAP54, SAP11, and TENGU effectors all alter plant development that resemble symptoms of phytoplasma-infected plants, but for SAP54 we have now shown that the alterations in plant development (leaf-like flowers) are not required for insect preference.

Targeting conserved plant proteins, such as MTFs and TCPs by phytoplasma effector proteins may enable the phytoplasma parasites to infect a broad range of plant species. The 26S proteasome shuttle proteins RAD23 are also conserved among plant species (Vierstra, 2009). Compatibility of phytoplasmas with multiple plant species is likely essential given that AY-WB phytoplasma and related parasites are transmitted by polyphagous insect species, such as leafhoppers of the genus *Macrosteles* (Lee et al., 2004; Weintraub and Beanland, 2006). Because these insects readily feed on many plant species, phytoplasmas will increase their fitness if they can modulate these plants to increase attraction and colonization of insect vectors. In agreement with this, SAP54 homologs are found in diverse phyllody-inducing phytoplasmas that infect a wide range of plant species (Bertaccini, 2007; MacLean et al., 2011; Maejima et al., 2014). Thus, generalist parasites, especially those dependent on alternative hosts for transmission, could gain fitness benefits via interfering with conserved host processes.

It is often argued that phenotypes induced by parasites have a direct positive relationship with parasite fitness (Gould and Lewontin, 1979; Dawkins, 1982, 1990). For example, frogs infected with Ribeiroia trematode species lose their legs and it is thought that this enhances the likelihood of these frogs to be eaten by birds, which are the secondary hosts (or vectors) of the trematodes. Because the trematodes depend on the birds for transmission to frogs (trematode-infested bird excretions get into snails, which are then eaten again by frogs), the trematode-induced alterations of frog morphology is seen as an adaptive parasite-induced phenotype, which has the primary role of enhancing trematode spread. Our hypothesis that leaf-like flowers promote insect vector attraction was argued from an adaptionist point of view, which was reinforced by our finding that SAP54 interaction with RAD23 is required for both leaf-like flower formation and insect vector attraction (MacLean et al., 2014). However, an alternative non-adaptionist view is that parasite-induced phenotypes are not always adaptive and could be side effects or secondary phenomena that have neutral effects on (parasite) fitness (Gould and Lewontin, 1979). In agreement with this, our results suggest that the formation of leaf-like flowers may be a phenotype that is secondary or a side effect to the primary role of SAP54 and phytoplasmas to attract leafhoppers (**Figure 6**). Therefore, leaf-like flowers may be an example of a non-adaptive parasite-induced phenotype.

## Author Contributions

ZO designed the experiments, carried out all experimental work, performed data analysis and wrote the manuscript; SH initiated the project, coordinated the study and wrote the manuscript. All authors give final approval for publication.

## Conflict of Interest Statement

The authors declare that the research was conducted in the absence of any commercial or financial relationships that could be construed as a potential conflict of interest.

## References

[B1] Artero R. Furlong E. E. Beckett K. Scott M. P. Baylies M. (2003). Notch and Ras signaling pathway effector genes expressed in fusion competent and founder cells during *Drosophila* myogenesis. *Development* 130 6257–6272. 10.1242/dev.00843 14602676

[B2] Bai F. Reinheimer R. Durantini D. Kellogg E. A. Schmidt R. J. (2012). TCP transcription factor, BRANCH ANGLE DEFECTIVE 1 (BAD1), is required for normal tassel branch angle formation in maize. *Proc. Natl. Acad. Sci. U.S.A.* 109 12225–12230. 10.1073/pnas.1202439109 22773815 PMC3409762

[B3] Berdoy M. Webster J. P. Macdonald D. W. (2000). Fatal attraction in rats infected with Toxoplasma gondii. *Proc. R. Soc. B-Biol. Sci.* 267 1591–1594. 10.1098/rspb.2000.1182 PMC1690701 11007336

[B4] Bertaccini A. (2007). Phytoplasmas: diversity, taxonomy, and epidemiology. *Front. Biosci.* 12:673–689. 10.2741/2092 17127328

[B5] Cezilly F. Favrat A. Perrot-Minnot M. J. (2013). Multidimensionality in parasite-induced phenotypic alterations: ultimate versus proximate aspects. *J. Exp. Biol.* 216 27–35. 10.1242/jeb.074005 23225864

[B6] Dawkins R. (1982). *The Extended Phenotype: The Long Reach of the Gene*. Oxford: Oxford University Press.

[B7] Dawkins R. (1990). Parasites, desiderata lists and the paradox of the organism. *Parasitology* 100 S63–S73. 10.1017/S0031182000073029 2235064

[B8] Dawkins R. (2004). Extended phenotype - But not too extended. A reply to Laland, Turner and Jablonka. *Biol. Philos.* 19 377–396. 10.1023/B:BIPH.0000036180.14904.96

[B9] Erb M. Meldau S. Howe G. A. (2012). Role of phytohormones in insect-specific plant reactions. *Trends Plant Sci.* 17 250–259. 10.1016/j.tplants.2012.01.003 22305233 PMC3346861

[B10] Frost K. E. Esker P. D. Van Haren R. Kotolski L. Groves R. L. (2013). Factors influencing aster leafhopper (Hemiptera: Cicadellidae) abundance and aster yellows phytoplasma infectivity in Wisconsin carrot fields. *Environ. Entomol.* 42 477–490. 10.1603/EN12239 23726057

[B11] Frost K. E. Willis D. K. Groves R. L. (2011). Detection and variability of aster yellows phytoplasma titer in its insect vector, *Macrosteles quadrilineatus* (Hemiptera: Cicadellidae). *J. Econ. Entomol.* 104 1800–1815. 10.1603/EC11183 22299339

[B12] Giron D. Glevarec G. (2014). Cytokinin-induced phenotypes in plant-insect interactions: learning from the bacterial world. *J. Chem. Ecol.* 40 826–835. 10.1007/s10886-014-0466-5 24944001

[B13] Gould S. J. Lewontin R. C. (1979). Spandrels of san-marco and the panglossian paradigm - a critique of the adaptationist program. *Proc. R. Soc. Ser. B Biol. Sci.* 205 581–598. 10.1098/rspb.1979.0086 42062

[B14] Gregis V. Andres F. Sessa A. Guerra R. F. Simonini S. Mateos J. L. (2013). Identification of pathways directly regulated by SHORT VEGETATIVE PHASE during vegetative and reproductive development in *Arabidopsis*. *Genome Biol.* 14:R56 10.1186/gb-2013-14-6-r56 PMC3706845 23759218

[B15] Hoover K. Grove M. Gardner M. Hughes D. P. McNeil J. Slavicek J. (2011). A gene for an extended phenotype. *Science* 333 1401–1401. 10.1126/science.1209199 21903803

[B16] Hoshi A. Oshima K. Kakizawa S. Ishii Y. Ozeki J. Hashimoto M. (2009). A unique virulence factor for proliferation and dwarfism in plants identified from a phytopathogenic bacterium. *Proc. Natl. Acad. Sci. U.S.A.* 106 6416–6421. 10.1073/pnas.0813038106 19329488 PMC2669400

[B17] Immink R. G. H. Pose D. Ferrario S. Ott F. Kaufmann K. Valentim F. L. (2012). Characterization of SOC1’s central role in flowering by the identification of its upstream and downstream regulators. *Plant Physiol.* 160 433–449. 10.1104/pp.112.202614 22791302 PMC3440217

[B18] Johnson P. T. J. Sutherland D. R. Kinsella J. M. Lunde K. B. (2004). Review of the trematode genus *Ribeiroia* (Psilostomidae): ecology, life history and pathogenesis with special emphasis on the amphibian malformation problem. *Adv. Parasitol.* 57 191–253. 10.1016/S0065-308X(04)57003-3 15504539

[B19] Kallenbach M. Bonaventure G. Gilardoni P. A. Wissgott A. Baldwin I. T. (2012). Empoasca leafhoppers attack wild tobacco plants in a jasmonate-dependent manner and identify jasmonate mutants in natural populations. *Proc. Natl. Acad. Sci. U.S.A.* 109 E1548–E1557. 10.1073/pnas.1200363109 22615404 PMC3386116

[B20] Lee I. M. Gundersen-Rindal D. E. Davis R. E. Bottner K. D. Marcone C. Seemuller E. (2004). ‘Candidatus *Phytoplasma asteris*’, a novel phytoplasma taxon associated with aster yellows and related diseases. *Int. J. Syst. Evol. Microbiol.* 54 1037–1048. 10.1099/ijs.0.02843-0 15280267

[B21] Lemaitre B. Nicolas E. Michaut L. Reichhart J. M. Hoffmann J. A. (1996). The dorsoventral regulatory gene cassette spatzle/Toll/cactus controls the potent antifungal response in *Drosophila* adults. *Cell* 86 973–983. 10.1016/S0092-8674(00)80172-5 8808632

[B22] Lu Y. T. Li M. Y. Cheng K. T. Tan C. M. Su L. W. Lin W. Y. (2014). Transgenic plants that express the phytoplasma effector SAP11 show altered phosphate starvation and defense responses. *Plant Physiol.* 164 1456–1469. 10.1104/pp.113.229740 24464367 PMC3938633

[B23] MacLean A. M. Orlovskis Z. Kowitwanich K. Zdziarska A. M. Angenent G. C. Immink R. G. H. (2014). Phytoplasma effector SAP54 hijacks plant reproduction by degrading MADS-box proteins and promotes insect colonization in a RAD23-dependent manner. *PLoS Biol.* 12:e1001835 10.1371/journal.pbio.1001835 PMC3979655 24714165

[B24] MacLean A. M. Sugio A. Makarova O. V. Findlay K. C. Grieve V. M. Toth R. (2011). Phytoplasma effector SAP54 induces indeterminate leaf-like flower development in *Arabidopsis* plants. *Plant Physiol.* 157 831–841. 10.1104/pp.111.181586 21849514 PMC3192582

[B25] Maejima K. Iwai R. Himeno M. Komatsu K. Kitazawa Y. Fujita N. (2014). Recognition of floral homeotic MADS domain transcription factors by a phytoplasmal effector, phyllogen, induces phyllody. *Plant J.* 78 541–554. 10.1111/tpj.12495 24597566 PMC4282529

[B26] Mandel M. A. Gustafson-Brown C. Savidge B. Yanofsky M. F. (1992). Molecular characterization of the *Arabidopsis* floral homeotic gene APETALA1. *Nature* 360 273–277. 10.1038/360273a0 1359429

[B27] Minato N. Himeno M. Hoshi A. Maejima K. Komatsu K. Takebayashi Y. (2014). The phytoplasmal virulence factor TENGU causes plant sterility by downregulating of the jasmonic acid and auxin pathways. *Sci. Rep.* 4:7399 10.1038/srep07399 PMC4261181 25492247

[B28] Naessens E. Dubreuil G. Giordanengo P. Baron O. L. Minet-Kebdani N. Keller H. (2015). A secreted MIF cytokine enables aphid feeding and represses plant immune responses. *Curr. Biol.* 25 1898–1903. 10.1016/j.cub.2015.05.047 26119751

[B29] Poulin R. (1995). “Adaptive” changes in the behaviour of parasitized animals: a critical review. *Int. J. Parasitol.* 25 1371–1383. 10.1016/0020-7519(95)00100-X 8719948

[B30] Poulin R. (2013). Parasite manipulation of host personality and behavioural syndromes. *J. Exp. Biol.* 216 18–26. 10.1242/jeb.073353 23225863

[B31] Schafer M. Meza-Canales I. D. Navarro-Quezada A. Brutting C. Vankova R. Baldwin I. T. (2015). Cytokinin levels and signaling respond to wounding and the perception of herbivore elicitors in *Nicotiana attenuata*. *J. Integr. Plant Biol.* 57 198–212. 10.1111/jipb.12227 24924599 PMC4286249

[B32] Schommer C. Palatnik J. F. Aggarwal P. Chetelat A. Cubas P. Farmer E. E. (2008). Control of jasmonate biosynthesis and senescence by miR319 targets. *PLoS Biol.* 6:e230 10.1371/journal.pbio.0060230 PMC2553836 18816164

[B33] Sugio A. Kingdom H. N. MacLean A. M. Grieve V. M. Hogenhout S. A. (2011a). Phytoplasma protein effector SAP11 enhances insect vector reproduction by manipulating plant development and defense hormone biosynthesis. *Proc. Natl. Acad. Sci. U.S.A.* 108 E1254–E1263. 10.1073/pnas.1105664108 22065743 PMC3228479

[B34] Sugio A. MacLean A. M. Kingdom H. N. Grieve V. M. Manimekalai R. Hogenhout S. A. (2011b). Diverse targets of *Phytoplasma* effectors: from plant development to defense against insects. *Annu. Rev. Phytopathol.* 49 175–195. 10.1146/annurev-phyto-072910-095323 21838574

[B35] Sugio A. MacLean A. M. Hogenhout S. A. (2014). The small phytoplasma virulence effector SAP11 contains distinct domains required for nuclear targeting and CIN-TCP binding and destabilization. *New Phytol.* 202 838–848. 10.1111/nph.12721 24552625 PMC4235307

[B36] Vierstra R. D. (2009). The ubiquitin-26S proteasome system at the nexus of plant biology. *Nat. Rev. Mol. Cell Biol.* 10 385–397. 10.1038/nrm2688 19424292

[B37] Wang X. Gao J. Zhu Z. Dong X. Wang X. Ren G. (2015). TCP transcription factors are critical for the coordinated regulation of ISOCHORISMATE SYNTHASE 1 expression in *Arabidopsis thaliana*. *Plant J.* 82 151–162. 10.1111/tpj.12803 25702611

[B38] Weigel D. Alvarez J. Smyth D. R. Yanofsky M. F. Meyerowitz E. M. (1992). LEAFY controls floral meristem identity in *Arabidopsis*. *Cell* 69 843–859. 10.1016/0092-8674(92)90295-N 1350515

[B39] Weintraub P. G. Beanland L. (2006). Insect vectors of phytoplasmas. *Annu. Rev. Entomol.* 51 91–111. 10.1146/annurev.ento.51.110104.151039 16332205

[B40] Wilson D. C. Carella P. Isaacs M. Cameron R. K. (2013). The floral transition is not the developmental switch that confers competence for the *Arabidopsis* age-related resistance response to *Pseudomonas syringae* pv. tomato. *Plant Mol. Biol.* 83 235–246. 10.1007/s11103-013-0083-7 23722504 PMC3777159

