# Supplementary figures and images for: A Bacterial Parasite Effector Mediates Insect Vector Attraction in Host Plants Independently of Developmental Changes
Source: Front Plant Sci. 2016 Jun 23;7:885. doi: 10.3389/fpls.2016.00885 (PMC4917533; doi:10.3389/fpls.2016.00885)

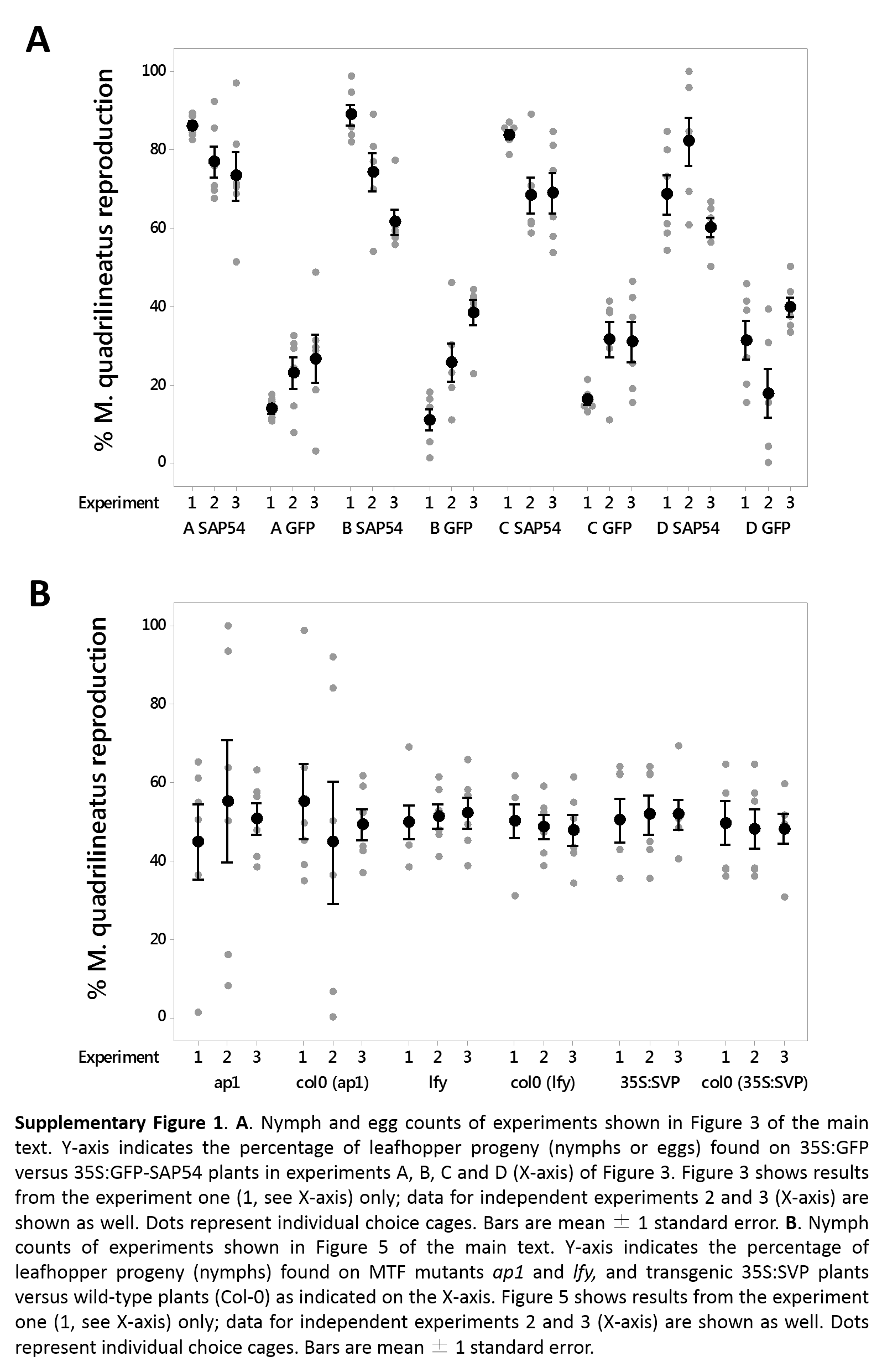

Supplement: Supplementary file 1 [file Image_1.TIF]
